# Transplantation of Human Induced Pluripotent Stem Cell-Derived Retinal Pigment Epithelium in a Swine Model of Geographic Atrophy

**DOI:** 10.3390/ijms221910497

**Published:** 2021-09-28

**Authors:** Anna Duarri, Eduardo Rodríguez-Bocanegra, Gema Martínez-Navarrete, Marc Biarnés, Miriam García, Lucía Lee Ferraro, Bernd Kuebler, Begoña Aran, Elisabeth Izquierdo, Eli Aguilera-Xiol, Ricardo P. Casaroli-Marano, Esteve Trias, Eduardo Fernandez, Ángel Raya, Anna Veiga, Jordi Monés

**Affiliations:** 1 Program for Clinical Translation of Regenerative Medicine in Catalonia–P-CMR[C], Bellvitge Biomedical Research Institute (IDIBELL), Hospitalet de Llobregat, 08908 Barcelona, Spain; anna.duarri@vhir.org (A.D.); bkuebler@idibell.cat (B.K.); baran@idibell.cat (B.A.); araya@idibell.cat (Á.R.); 2 National Stem Cell Bank-Barcelona Node, Biomolecular and Bioinformatics Resources Platform PRB2, ISCIII, IDIBELL, Hospitalet de Llobregat, 08908 Barcelona, Spain; 3 Ophthalmology Research Group, Vall d’Hebron Institut de Recerca (VHIR), 08036 Barcelona, Spain; 4 Barcelona Macula Foundation: Research for Vision, 08022 Barcelona, Spain; eduardo.rodriguez-bocanegra@med.uni-tuebingen.de (E.R.-B.); mbiarnes@barcelonamaculafound.org (M.B.); mgarcia@barcelonamaculafound.org (M.G.); lferraro@barcelonamaculafound.org (L.L.F.); 5 Institut de la Màcula, Centro Médico Teknon, 08022 Barcelona, Spain; 6 Networking Research Center of Bioengineering, Biomaterials and Nanomedicine (CIBER-BBN), 28029 Madrid, Spain; gemamartineznavarrete@gmail.com (G.M.-N.); e.fernandez@umh.es (E.F.); 7 Institute of Bioengineering, Universidad Miguel Hernández, 03202 Alicante, Spain; 8 Specific Pig S.L, 08820 Barcelona, Spain; elisabeth.izquierdo@specipig.com (E.I.); eli.aguilera@specipig.com (E.A.-X.); 9 Banc de Sang i Teixits (BST), Institute of Biomedical Research (IIB-Sant Pau), 08025 Barcelona, Spain; rcasaroli@ub.edu; 10 Department of Surgery, School of Medicine and Health Science, Hospital Clinic de Barcelona, University of Barcelona, 08036 Barcelona, Spain; 11 LEITAT Technological Center, 08005 Barcelona, Spain; etrias@leitat.org; 12 Advanced Therapies Unit, Hospital Clínic de Barcelona, 08005 Barcelona, Spain; 13 Institució Catalana de Recerca i Estudis Avançats (ICREA), 08010 Barcelona, Spain

**Keywords:** age-related macular degeneration (AMD), geographic atrophy, pig, animal model, stem cells, iPSC, RPE, retina, regenerative medicine, advanced cell therapy

## Abstract

Background: The aim of this study was to test the feasibility and safety of subretinal transplantation of human induced pluripotent stem cell (hiPSC)-derived retinal pigment epithelium (RPE) cells into the healthy margins and within areas of degenerative retina in a swine model of geographic atrophy (GA). Methods: Well-delimited selective outer retinal damage was induced by subretinal injection of NaIO_3_ into one eye in minipigs (*n* = 10). Thirty days later, a suspension of hiPSC-derived RPE cells expressing green fluorescent protein was injected into the subretinal space, into the healthy margins, and within areas of degenerative retina. In vivo follow-up was performed by multimodal imaging. Post-mortem retinas were analyzed by immunohistochemistry and histology. Results: In vitro differentiated hiPSC-RPE cells showed a typical epithelial morphology, expressed RPE-related genes, and had phagocytic ability. Engrafted hiPSC-RPE cells were detected in 60% of the eyes, forming mature epithelium in healthy retina extending towards the border of the atrophy. Histological analysis revealed RPE interaction with host photoreceptors in the healthy retina. Engrafted cells in the atrophic zone were found in a patchy distribution but failed to form an epithelial-like layer. Conclusions: These results might support the use of hiPSC-RPE cells to treat atrophic GA by providing a housekeeping function to aid the overwhelmed remnant RPE, which might improve its survival and therefore slow down the progression of GA.

## 1. Introduction

Age-related macular degeneration (AMD) is the most common cause of blindness in people older than 50 years in industrialized countries, and affects between 14.9 and 21.5 million people in Europe alone [1]. AMD is a degenerative, chronic, and progressive disease involving the retinal pigment epithelium (RPE) and choroid, which trigger the degeneration of the outer retina in the macula region of the eye [2]. The etiology of AMD is multifactorial and includes multiple genetic and environmental factors [3]. There are two forms of AMD—exudative (wet, neovascular) or atrophic (dry). The first one is related to the development of choroidal neovascularization and accounts for about 20% of all cases of AMD. Late-stage dry AMD can manifest as geographic atrophy (GA), which affects the macular region of the retina, causing progressive and irreversible loss of central vision due to the degeneration of retinal pigment epithelium (RPE) and photoreceptor cells, and accounts for 80% of all cases. At present, while there are the intravitreal injections of anti-VEGF factors used in the management of wet AMD, there is no effective curative treatment or preventive therapy for the dry form of this disease. Once RPE and photoreceptor cells are lost, cell replacement or prosthetic devices are the only potential therapeutic options [4,5]. 

Despite the evident degeneration of RPE and photoreceptors in AMD, the inner retina and neural connections seem to be preserved, to some extent, for a limited time, supporting the realistic potential of cell-based therapy to restore vision [6]. Human pluripotent stem cells—both embryonic (hESC) and induced (hiPSC)—have the ability to self-renew and to differentiate into all retinal cell types needed for retinal regeneration [7]. Indeed, hESC-derived RPE cells are currently being investigated in 20 early-phase human clinical trials to treat retinopathies (reviewed in [8,9,10,11]) (https://clinicaltrials.gov/ queried on 1 March 2019). Follow-up studies have shown that transplantation of RPE sheets is tolerable, but requires very invasive surgical procedures with associated safety concerns. By contrast, transplantation of RPE cell suspensions requires minimal surgery, making this procedure more appealing for clinical applications and more suitable for wider and easier adoption by the ophthalmological community [12,13,14,15,16,17]. Nevertheless, more pre-clinical research is needed to better understand how RPE cell suspensions survive and integrate, mature, and interact with host photoreceptors, particularly those derived from hiPSC.

Due to their similar dimensions and anatomy, large animals can play important roles in the development and adaptation of human surgical techniques and are good pre-clinical models for cell replacement treatments [18]. In particular, the pig eye and retina resemble their human counterparts in terms of size and shape, number, and distribution of photoreceptors, vasculature, and function [19]. Accordingly, several transgenic pig models have been developed to study retinitis pigmentosa [20,21,22,23,24]; however, the disease progression is slow, which hinders the evaluation of cell therapy benefits, and the models are expensive to maintain. To overcome these limitations and to bypass the genetic component, and also the aging and environmental factors that underlie retinal diseases, we have developed a pig model of well-delimited outer retinal degeneration through localized subretinal injection of sodium iodate (NaIO_3_), which resembles the selective degeneration of RPE or outer retina in humans, with special interest in two major aspects of human GA associated with the dry form of AMD, damage of outer retinal layers with preservation of the inner layers, and lesions will well-defined distinct margins between the atrophic area and the healthy area [25]. Other, less well-delimited, models have been established, such as intravenous injection of iodoacetic acid [26], light injury [27,28,29], scraping injury [29,30], or high-pressure injury [30], and these models have been used in studies of allogenic and xenogenic cell transplantation in healthy or degenerated porcine retinas using either porcine or human fetal neuroretina cells or sheets [27,29,30,31,32], mouse retinal progenitor cells [28], hESC-derived RPE monolayer on biocompatible scaffolds [33,34], or porcine iPSC-derived RPE and retinal progenitor cells [26,35]. The lack of pre-clinical studies in swine models involving injection of subretinal cell suspensions of human iPSC-derived retinal cells prompted us to test whether hiPSC-derived RPE cell transplantation in a model mimicking end-stage AMD might be a potential approach to treat this disease. 

Here, we investigated the feasibility and safety, survival, and integration of subretinal xenotransplantation of hiPSC-RPE cell suspensions in a NaIO_3_-induced GA model in miniature pigs. 

## 2. Results

### 2.1. Generation and Characterization of hiPSC-RPE Cells

**Figure 1:**
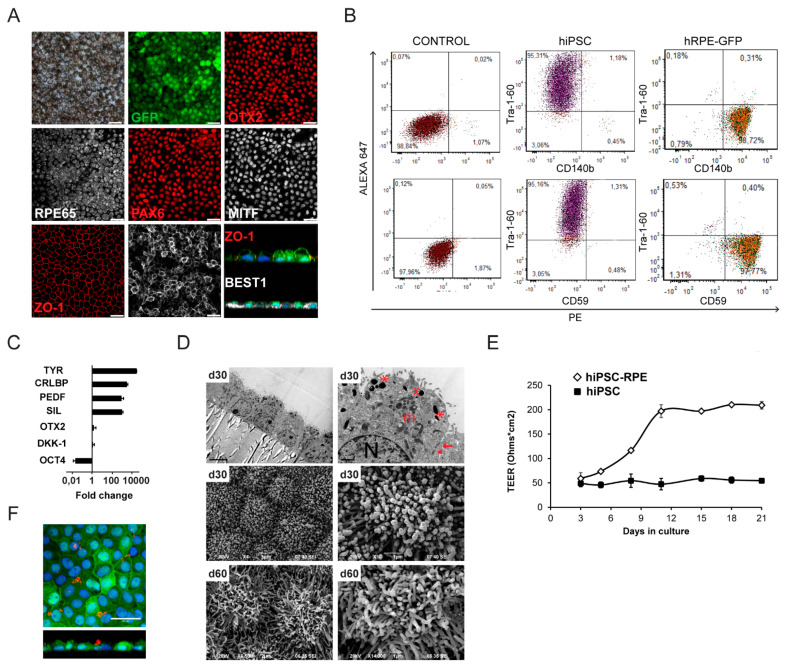
Characterization of hiPSC-derived RPE cells expressing GFP. (**A**) Representative bright-field and immunostaining images showing pigmented hiPSC-RPE at 30 days in culture and the expression of endogenous GFP and RPE markers: BEST-1 (bestrophin-1), ZO-1 (zonula occludens 1), PAX6 (paired box 6), MITF (microphthalmia-associated transcription factor), OTX2 (orthodenticle homeobox 2), and RPE65 (anti-retinal pigment epithelium-specific 65 kDa protein). hiPSC-RPE cells are negative for OCT4 (data not shown). Scale bars: 25 μm. Representative x–z scans of polarized hiPSC-RPE cells (green), apical ZO-1 (red), and basolateral bestrophin (white). Cell nuclei were stained with DAPI (blue). (**B**) Quantitative flow cytometry analysis of hiPSC and hPRE-GFP cells with surface markers Tra-1-60 (undifferentiated cells), CD140b, and CD59 (RPE-specific), and the appropriate isotype controls. The numbers in the corners show the percentage of stained cells in this gate. (**C**) Gene expression levels of OCT4, OTX2, SIL (silver), CRLBP (cellular retinal aldehyde-binding protein), PEDF (pigment epithelium-derived factor), and TYR (tyrosinase) in hiPSC-RPE cells by qPCR. Values are normalized to GAPDH and relative to hiPSC, and are expressed as 2^-ΔΔCt^ (log scale). (**D**) Representative electron microscopy images of hiPSC-RPE cells in Transwell insert cultures at days 30 and 60 in culture. TEM images (top) showing pigmented cuboidal epithelial monolayer, with apical microvilli, melanosomes (asterisk), basal nuclei (N), mitochondria (m), tight junctions (arrow), and adherent junctions (arrowheads). Scale bars: 5 μm (left), 1 μm (right). SEM images (bottom) showing the apical microvilli and polygonal cell morphology. Scale bars: 5 μm (middle left); 2 μm (bottom left); 1 μm (right). (**E**) Graph showing transepithelial electrical resistance (TEER) for hiPSC and hiPSC-RPE cells at indicated days in culture. (**F**) Confocal images showing phagocytosis of isolated TRITC-labeled bovine photoreceptor outer segments (POS, red) by hiPSC-RPE (green) in culture. Representative x–y and x–z images show total TRITC-labeled POS (red) taken up by the cells. Nuclei are stained with DAPI (blue). Scale bar: 25 µm.

To facilitate the identification of implanted hiPSC-RPE cells, we generated an hiPSC line (CBiPS30-4F-5) constitutively expressing GFP while maintaining pluripotency and normal karyotype (Supplementary Figure S1A,B). Differentiated and purified hiPSC-RPE cells were pigmented, maintained GFP expression, and had a hexagonal-shaped morphology (Figure 1A). Additionally, hiPSC-RPE cells expressed molecular markers of RPE such as PAX6 and OTX2, and the RPE cell-specific markers bestrophin-1 (basal membrane), MITF (optic vesicle), ZO-1 (tight junctions), SIL, RPE65 and CRALBP (visual cycle), PEDF (growth factors), and TYR (pigmentation), as detected by immunocytochemistry and qPCR (Figure 1A,C). Reassuringly, vertical confocal sections showed correct apical localization of ZO-1 and basolateral localization of bestrophin-1 in cells (Figure 1A). To assess the purity of hRPE-GFP cells, the hiPSC line together with hRPE-GFP derived from it were tested in parallel for Tra-1-60, a pluripotency marker, and for CD140b and CD59, RPE-specific markers, by flow cytometry (Figure 1B; Supplementary Figure S2). hRPE-GFP cells expressed CD140b and CD59 but not Tra-1-60, indicating the absence of undifferentiated cells in our culture. TEM analysis of the ultrastructure of hiPSC-RPE cells cultured in Transwell inserts showed a monolayer of pigmented cuboidal epithelial cells with cytoplasmic polarization, with nuclei located to the basolateral side, and microvilli, well-formed tight junctions, and melanin-containing melanosomes on the apical side (Figure 1C, top). Likewise, SEM analysis showed microvilli on the apical surface and a polygonal-shaped cellular morphology at days 30 and 60 in culture (Figure 1C, bottom). We also assessed the integrity of tight junctions’ dynamics in hiPSC-RPE cells by measuring transepithelial electrical resistance (TEER). TEER values were first detected at day 3 of Transwell culture and increased until day 11, when they reached a plateau at ~200 Ω·cm^2^, in contrast to hiPSC culture (Figure 1E). We then tested the functionality of hiPSC-RPE cells in vitro by analyzing their phagocytosis ability, finding that the cells phagocytosed bovine-purified TRITC-labeled POS (Figure 1F).

### 2.2. Generation of a Swine Model with Geographic Atrophy-like Features

**Figure 2:**
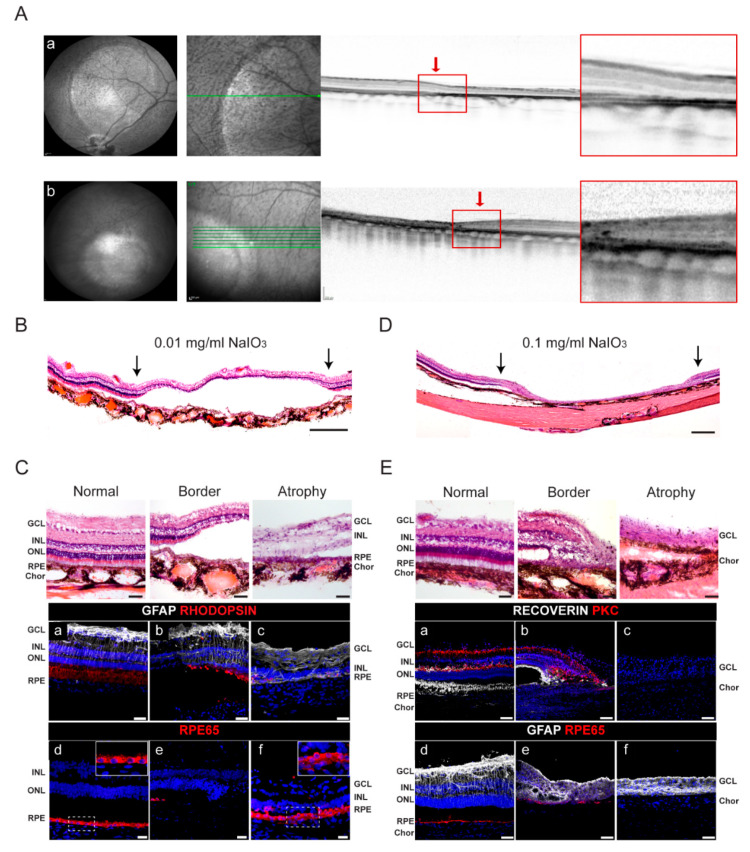
Selective and severe atrophies induced by subretinal injection of NaIO_3_ in porcine retinas. (**A**) Fundus porcine retinas at 1 month after NaIO_3_ subretinal injection (0.2 mL) at a concentration of 0.01 mg/mL (a). A well-demarcated round area is shown by IR fundus imaging after NaIO_3_ injection in pig 4. The 30° fundus IR image with overlying position of B-Scan (middle panel; green line) and cross-sectional SD-OCT B-scan demonstrate selective loss of the outer retinal layers. The outer plexiform layer and inner nuclear layer are directly on the damaged RPE, as occurs in GA in humans. (b) By contrast, a severe round atrophy is shown by IR and SD-OCT imaging after NaIO_3_ injection in pig 9. A transition zone (red arrows) is observed between the atrophic and healthy retina in SD-OCT in both (a) and (b). Red squares indicate the enlargement of the atrophic border. (**B**) Photomerge composition of hematoxylin and eosin (H&E) staining of the lesion induced with 0.01 mg/mL NaIO_3_ in pig 4. Arrows indicate the border of the atrophy. Scale bar: 200 µm. (**C**) (Top panels) Images of H&E staining showing different parts of porcine healthy and atrophic retina of the lesion induced with 0.01 mg/mL NaIO_3_. Scale bars: 50 µm. (Bottom panels) Immunofluorescence images of different areas (healthy (a,d), border (b,e), and mild atrophy (c,f)) labeled with glial fibrillary acidic protein (GFAP), retinal pigment epithelium-specific 65 kDa protein (RPE65) and rhodopsin. Insets in (**C** (d) and (f)) show an enlargement of the RPE layer stained with RPE65. Scale bars: 50 µm. (**D**) Photomerge composition of H&E staining of the lesion induced with 0.1 mg/mL NaIO_3_ in pig 8. Arrows indicate the border of the atrophy. Scale bar: 200 µm. (**E**) (Top panels) Images of H&E staining showing different parts of porcine healthy and atrophic retina of the lesion induced with 0.1 mg/mL NaIO_3_. Scale bars: 50 µm. (Bottom panels) Immunofluorescence images of the different zones of the eyes (healthy (a,d), border (b,e), and severe atrophy (c,f)) labeled with recoverin, protein kinase C alpha (PKC), GFAP, and RPE65. Scale bars: 50 µm. Nuclei are stained with DAPI (blue). NaIO_3_, sodium iodate; IR, infrared image; SD-OCT, spectral-domain optic coherence tomography; RPE, retinal pigment epithelium; ONL, outer nuclear layer; INL, inner nuclear layer; GCL, ganglion cell layer; GFP, green fluorescent protein; Chor, choroid.

Based on our previous work [25], we used the subretinal injection of NaIO_3_ to recapitulate the main features of GA in minipigs. Specifically, the right eye of 7 pigs (pigs 1–7; Table 1) was injected with the optimal concentration of NaIO_3_ (0.01 mg/mL). One month later, in vivo spectral domain optical coherence tomography (SD-OCT) analysis of these animals revealed selective atrophy of outer retinal layers (Figure 2A, a; Table 1; Supplementary Figure S3). We also explored whether more severe atrophy could affect the outcome of the RPE therapy. Thus, three pigs (pigs 8–10) were injected with a higher dose of NaIO_3_ (0.1 mg/mL), which resulted in severe atrophy with dramatic thinning of all retinal layers (Figure 2A, b; Supplementary Figure S3). In all pigs analyzed, outer retinal atrophy was maintained over time and a well-delimited transition zone between healthy and atrophic retinas was observed, thus mimicking the alterations seen in patients with advanced age-related macular degeneration and GA. 

Three animals, two with selective atrophy (animal 3 and 4) and one with severe atrophy (animal 8), were histologically analyzed because they showed no presence of hiPSC-RPE cells. Animals 3 and 4 showed selective atrophy, mostly limited to the integrity of RPE and the outer nuclear layer (ONL) (Figure 2B,C). In the selective atrophies, photoreceptors were absent from the border of the atrophy (Figure 2C, a–c), RPE cells were focally lost (Figure 2C, e), and the remaining RPE layer in the atrophic zone showed alterations in both cell morphology and organization compared with the healthy RPE layer (insets in Figure 2C, d,f), consistent with previous observations [25,36]. By contrast, the severe atrophy in pig 8 affected RPE, the ONL, and the inner nuclear layer (INL), with a well-delimited border (Figure 2D,E). Additionally, the bipolar cells (PKC-positive) in the INL and photoreceptors (recoverin-positive) were missing in the transition zone (Figure 2E, a–c). RPE cells were lost (as judged by the absence of the RPE65 signal), and this was accompanied by Bruch’s membrane (BM) atrophy (Figure 2D,E, d,f). We also observed that activated astrocytes (GFAP-positive) in the atrophic zone were densely packed (Figure 2E, d–f) and formed a membrane-like structure posterior to the photoreceptor segments in the subretinal space (Supplementary Figure S4A), likely due to the accumulation of junctions within glial processes, as has been described in patients with GA [37].

### 2.3. In Vivo Imaging of Transplanted hiPSC-RPE Cells in the Porcine Eyes

**Figure 3:**
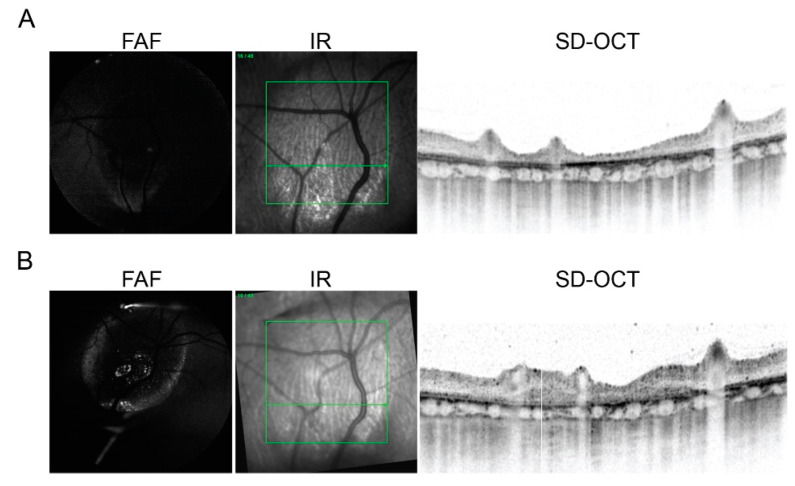
Representative image of FAF and SD-OCT of (**A**) prior to and (**B**) two weeks after hiPSC-RPE cell subretinal injection in pig 6. (**A**) The day before hiPSC-RPE cell injection, no evidence of hyper-autofluorescence due to GFP expression is observed on FAF, and a marked atrophy and thinning of all retinal layers is present on SD-OCT. (**B**) Fifteen days after the hiPSC-RPE cell injection, several foci of hyper-autofluorescence are observed inside the area of atrophy on FAF, but also an increase in retinal thickness as compared with (**A**). Green squares delineate scan area and green horizontal line show scan directions and level of right image FAF, fundus autofluorescence; GFP, green fluorescence protein; SD-OCT, spectral-domain optic coherence tomography; RPE, retinal pigment epithelium.

**Figure 4:**
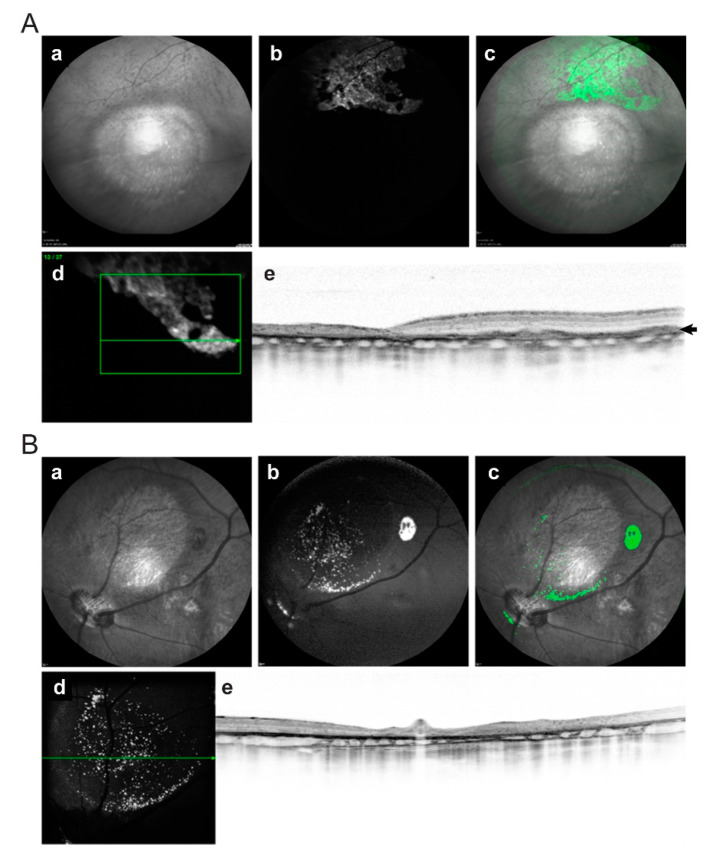
Multimodal fundus imaging showing the presence of hiPSC-RPE cells in healthy (**A**) and atrophic (**B**) retina in pigs 9 and 7 respectively, at 3 months post-injection. Well-demarcated round areas by IR fundus (a) four months after NaIO_3_ subretinal injection. FAF (b) showing hyper-autofluorescence generated by the GFP expression in subretinal injected hiPSC-RPE cells. Pseudo-colored images (c) from increased autofluorescence in FAF imaging using Fiji show widespread (**A**, c,d) and localized (**B**, c,d) hiPSC-RPE cells (in green) outside the atrophic area, but also inside the atrophy region (**B**, d). On FAF-guided SD-OCT, a well-organized RPE monolayer appears between native RPE and photoreceptors (**A**, e) as a new hyper-reflective layer of hiPSC-RPE cells in healthy retina. A granular distribution of the hiPSC-RPE cells (hyper-reflective dots) is shown in the outer retinal layers of the atrophic retina (**B**, e). Green squares delineate scan area and green horizontal line show scan directions and level. FAF, fundus autofluorescence; GFP, green fluorescence protein; NaIO_3_, sodium iodate; SD-OCT, spectral-domain optic coherence tomography; RPE, retinal pigment epithelium.

One month following NaIO_3_ injection, hiPSC-RPE cells were injected into the subretinal space of animals 1–7 with selective atrophy and animals 8 and 9 with severe atrophy (Table 1). Animal 10 was not transplanted because of the development of retinal detachment during the follow-up after atrophy induction, and animal 2 died two weeks after transplantation from causes unrelated to the procedure. To assess the traceability of the injected cells, in vivo post-operative follow-up was performed using SD-OCT, and fundus autofluorescence (FAF) and infrared (IR) imaging. SD-OCT images were acquired with simultaneous visualization of the FAF in the IR channel. Constitutive GFP expression of hiPSC-RPE cells was clearly detectable through FAF imaging as hyper-autofluorescence signals in pigs 1, 5, 6, 7, and 9 (60% of the animals) (Figure 3 and Figure 4; Table 1). In pigs 1 and 5, hyper-autofluorescence signals were only visible in the first ophthalmological exam after hiPSC-RPE cell injection, whereas in pigs 6, 7, and 9, the signals were maintained up to three months (latest time-point analyzed). Two weeks after cell injection, FAF imaging revealed a hyper-autofluorescence speckle in the atrophied area and its border (animal 6, compare Figure 3A (before cell injection) and Figure 3B (after cell injection)). Additionally, SD-OCT analysis showed a thickening of the outer retinal layer due to the new appearance of subretinal hyper-reflective material that might correspond to the transplanted hiPSC-RPE cells, both in the healthy retina area and within the atrophic zone (Figure 3B), and this was also observed in animals 1, 5, 6, 7, and 9. At three months, FAF imaging detected homogeneous signals of hyper-autofluorescence in healthy areas of the retina (animals 7 and 9, Figure 4A). Cells appeared well-organized in a monolayer, localized between the RPE and photoreceptors as a new hyper-reflective layer, as visualized by SD-OCT (Figure 4A, e). Conversely, the engraftment of hiPSC-RPE cells in atrophic areas occurred in a much more punctual manner, and was predominantly at the atrophy border (Figure 4B).

### 2.4. Post-Mortem Analysis of Retinas Transplanted with hiPSC-RPE Cells

Animal 1 was euthanized at 6 months, animal 2 died and was analyzed at 2 weeks, and the remaining animals were euthanized at 3 months (Table 1). Surviving hiPSC-RPE cells were found engrafted in areas of healthy retina in 5 animals (1, 2, 5, 6, and 9), and at the margins of the atrophy and/or within the atrophied retina in 4 animals (1, 2, 7, and 9) (Table 1).

**Figure 5:**
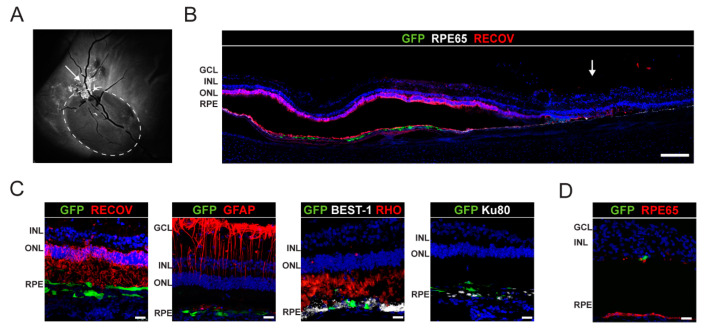
Analysis of engrafted hiPSC-RPE cells in the host porcine retinas at two weeks post-transplantation in pig 4. (**A**) FAF shows hyper-fluorescence corresponding to hiPSC-RPE cells located in an area covering the healthy retina, the limiting margin, and a small part of the atrophy (circle denotes the atrophic zone). (**B**) Immunohistochemical analysis shows hiPSC-RPE cell integration in healthy retinas close to the margin of the atrophy stained with RPE65 and recoverin (RECOV) (white arrow indicates the atrophy border). Scale bar: 250 µm. (**C**) Confocal images show engrafted hiPSC-RPE cells (GFP in green) in healthy retina, stained with recoverin and rhodopsin (RECOV and RHO), expressing bestrophin-1 (BEST-1) and Ku80 (human nuclear marker). Astrocytes show normal morphology (GFAP). Scale bars: 25 µm. (**D**) Immunofluorescence images of hiPSC-RPE integration in atrophic areas stained with RPE65. Scale bars: 25 µm. Cell nuclei were stained with DAPI (blue). FAF, fundus autofluorescence; RPE, retinal pigment epithelium; OS, outer segments; ONL, outer nuclear layer; INL, inner nuclear layer; GCL, ganglion cell layer; Chor, choroid; GFP, green fluorescent protein.

Post-mortem analysis of animal 2 at 2 weeks following transplantation revealed hyper-autofluorescence corresponding to engrafted hiPSC-RPE cells in an area covering the healthy retina, the limiting border, and a small part of the atrophy area (Figure 5A, circle denotes the atrophic zone, and Figure 5B). Within the healthy retina, GFP-positive cells exhibited an elongated cell morphology, interacted with POS (recoverin and rhodopsin), and expressed the human nuclear antigens Ku80 and bestrophin-1. We also found isolated GFP+ cells within the atrophied area (Figure 5D).

**Figure 6:**
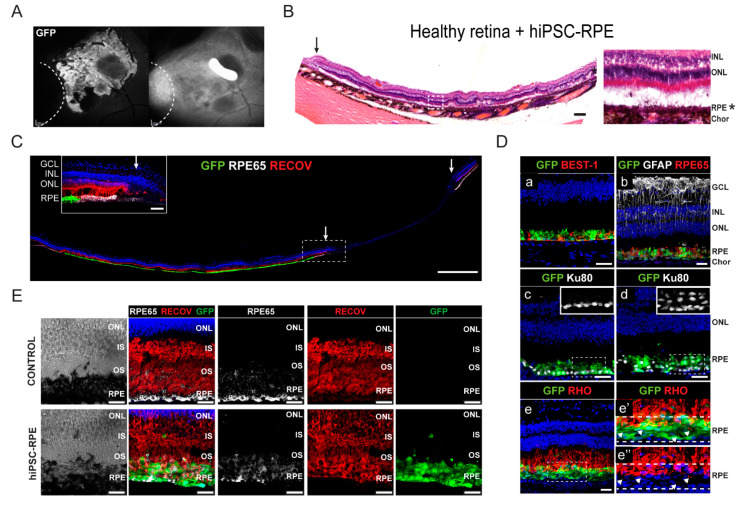
Analysis of engrafted hiPSC-RPE cells in healthy porcine retinas at three months post-transplantation in pigs 6 and 9. (**A**) Post-fixed FAF and IR of transplanted hiPSC-RPE cells in healthy retinas near the atrophy border. Atrophy is marked with a dashed line. (**B**) Compositions of hematoxylin and eosin (H&E) staining showing transplanted hiPSC-RPE cells (asterisk) in healthy retina. Arrow indicates the atrophy border. Scale bar: 400 µm. White dashed square indicates a higher magnification image (right panel) showing the transplanted hiPSC-RPE cell layer (asterisk). Note that green fluorescence could not be seen after H&E staining. (**C**) Confocal composition of integrated hiPSC-RPE cell layer in the healthy retina extending towards the transition zone of the atrophic area in pig 9 immunolabeled with RPE65 and recoverin (RECOV). Scale bars: 1000 µm. Inset shows magnification of the margin of the atrophy indicated by a dashed square. Arrows indicate atrophy border. Scale bars: 50 µm. (**D**) Immunohistochemical staining of engrafted hiPSC-RPE cells in healthy retina showing the expression of RPE-specific markers bestrophin-1 (BEST-1) and RPE65 (a,b). Retinal glia was stained with GFAP. The human nuclear antigen Ku80 was used to detect human cells corresponding to transplanted hiPSC-RPE cells (c,d, insets show magnification of Ku80+ nuclei). Photoreceptor cells were detected by rhodopsin (RHO) marker (e). Enlargement RHO (e′,e″) immunofluorescence images showing contact between hiPSC-RPE cells and photoreceptor outer segments revealing numerous immunolabeled phagosomes (white arrowheads). Dashed lines indicate the GFP+ hiPSC-RPE layer. Scale bars: 25 µm. (**E**) Bright-field images of control and transplanted retinas (left panels). Immunohistochemical staining of control retina and transplanted retina with engrafted hiPSC-RPE cells showing the expression of RPE-specific marker RPE65 and photoreceptor marker recoverin (RECOV) (right panels). Scale bars: 25 µm. Cell nuclei were stained with DAPI (blue). FAF, fundus autofluorescence; IR, infrared image; RPE, retinal pigment epithelium; ONL, outer nuclear layer; INL, inner nuclear layer; GCL, ganglion cell layer; Chor, choroid; GFP, green fluorescent protein.

**Figure 7:**
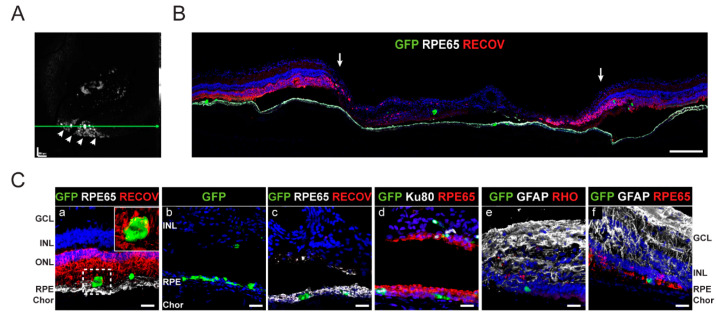
Analysis of hiPSC-RPE cell engraftment in atrophic porcine retinas in pigs 7 and 1. (**A**) In vivo FAF corresponding to the atrophic area transplanted with hiPSC-RPE cells in pig 7 at three months post-transplantation shows punctual integration of cells that are more prominent in the atrophic border (white arrowheads). Scale bar: 600 µm. (Green horizontal line show OCT scan level, not shown)(**B**) Immunostaining composition of the atrophic area stained with RPE65 and recoverin (RECOV) corroborates punctual engraftment of hiPSC-RPE cells. Arrows indicate atrophy borders. Scale bar: 250 µm. (**C**) Detailed confocal images of hiPSC-RPE cells engrafted in the atrophy margin (a) located in the RPE layer (RPE65) interacting with photoreceptors (RECOV), and in the atrophy (b–e) within the RPE layer (RPE65) and the remaining INL. Transplanted hiPSC-RPE expressed human nuclei marked with Ku80 (d). Astrocyte disorganization is shown by GFAP (e,f) and the lack of photoreceptor by RECOV (c) and rhodopsin (RHO) (e). Scale bars: 25 µm. At six months, few hiPSC-RPE cells integrated in the RPE layer (RPE65) within the atrophic area (f). Scale bars: 50 µm. Cell nuclei were stained with DAPI (blue). FAF, fundus autofluorescence; RPE, retinal pigment epithelium; ONL, outer nuclear layer; INL, inner nuclear layer; GCL, ganglion cell layer; Chor, choroid; GFP, green fluorescent protein.

Surviving hiPSC-RPE cells were found engrafted in areas of the healthy retina at 3 months in 3 animals (5, 6, and 9), and at the margins of the atrophy, or in the atrophic retina, in 2 animals (9 and 7; Figure 6 and Figure 7, respectively; Table 1). Moreover, we could detect GFP-positive cells in the vitreous of two pigs (pigs 5 and 7; Supplementary Figure S4B). hiPSC-RPE cells formed a well-organized epithelium-like layer (Figure 6B,C). Histological analysis confirmed normal gross retinal morphology in areas containing engrafted cells, with no neoplastic structures (Figure 6B). Surviving hiPSC-RPE cells were pigmented at levels similar to those of host RPE cells (asterisk in Figure 6B,E. Transplanted cells extended close to the limiting border of the atrophy (Figure 6C, arrow indicates the atrophy border), expressed bestrophin-1 and RPE65 (Figure 6D, a,b; Supplementary Figure S4C), and formed both mononuclear and multinuclear layers with a nearly normal pattern (insets in Figure 6C, c,d). Moreover, engrafted hiPSC-RPE cells showed numerous immunolabeled phagosomes (recoverin-positive or rhodopsin-positive) in cross-sections, indicating a potential interaction with host photoreceptors (Figure 6D, e,e′,e″ Figure 6E) [38].

FAF imaging of pig 7 revealed several hiPSC-RPE cells engrafted in a punctuated manner within the atrophic zone, accumulating in the vicinity of the border with healthy retina (Figure 7A, arrowheads). Immunohistochemical analysis of cryosections revealed the discrete integration of transplanted cells in the RPE layer along the atrophy and in the margins (Figure 7B,C, a). We also observed hiPSC-RPE cells engrafted within the remaining INL (Figure 7C, b–d), although in some areas, hiPSC-RPE cells formed epithelial-like structures (Figure 7C, b).

## 3. Discussion

Here, we used a miniature pig model of GA that resembles some advanced atrophic form of human AMD features to study the subretinal hiPSC-derived RPE cell transplantation as a potential therapy to treat GA. Our main objective was to assess the approach’s feasibility, and the safety, survival, and potential of cells to integrate within the tissue, particularly at the margins of the localized well-defined GA lesions. Much progress has been achieved in the treatment of the exudative form of AMD, which was once considered to be a devastating condition. However, there is no current treatment for GA, the atrophic form of AMD, and several recent trials have failed to demonstrate efficacy against this progressing blinding condition [39]. RPE cell degeneration and death is prevalent in GA and, within a few years, leads to very severe impairment of visual function and legal blindness [40]. Attempts to regenerate or rescue already dead tissue appear to be a challenge because of the extremely sophisticated tridimensional network of synapses of each retinal cell to numerous different types of surrounding cells. Results of cell transplantation studies show high variability of cell survival and a lack of integration in atrophic retinas, which might be caused by factors that trigger graft rejection, either by autoimmune response [41], acquired immune response [35], or by environmental factors associated with the degenerative retinal state. Moreover, damage to RPE and BM layers implies a disruption of the retinal-blood barrier, which is potentially more susceptible to inflammatory response [42]. More importantly, perhaps, photoreceptor loss initiates a cascade of negative neuronal remodeling of the retina and cell death, in addition to reactive gliosis of the astrocytes and Müller cells and the formation of scar tissue [43]. Accordingly, one of the theoretical aims of stem cell-based therapy with pluripotent stem cell-derived retinal cells—integrating into and rewiring host cells—might be a challenging endeavor. Another potential beneficial mechanism would rely on neurotrophic factor secretion without actual stem-cell integration into the host tissue. A third potential mechanism of benefit would be stem cell survival and limited integration into the host tissue without true rewiring, which could assist the overwhelmed host cells with their housekeeping functions. In any case, regenerative effects appear to be highly unlikely within areas of long-standing well-established atrophy due to the aforementioned reasons, especially due to neuronal remodeling. Therefore, the interest is focused on exploring if regenerative processes may still occur at the margins of the atrophy, where the retinal network and microenvironment are partially preserved.

AMD is characterized by extracellular deposits, and a hallmark of the disease is the presence of different types of drusen [44,45], which have been demonstrated to be toxic for RPE cells [46]. In addition, it is known that cell-to-cell transmission of pathogenic proteins plays a role in neurodegenerative diseases [47]. In a similar line, a decline in the endo-lysosomal function within the RPE may lead to functional deficits and an increase of intracellular debris such as lipofuscin [48,49], which might overwhelm the capacity of RPE to remove metabolic debris from the visual cycle, ultimately leading to RPE cell death and atrophy [50,51]. Therefore, we hypothesize that RPE cells injected around and at the boundaries of the atrophy, where there are still viable cells, might play a role in assisting with housekeeping functions of the overwhelmed host RPE cells. This would not need the complex integration with photoreceptors that true regeneration would require, but only survival and a certain degree of integration to aid host RPE cells in removing the debris and deposits occurring in the setting of AMD. This might potentially help the host RPE cells to survive longer. The present study was designed with this rationale in mind; specifically, to evaluate whether hiPSC-RPE cells could survive and (partially) integrate into the retina of the margins of the atrophic areas.

The lack of pre-clinical studies on hiPSC-based therapies in swine models of end-stage AMD raised the question of whether hiPSC-RPE cells’ suspension could integrate into porcine eyes, either in the normal retina or in the induced GA. There are various techniques to induce GA models, such as laser-high-pressure or light-induced injuries, retinal scraping, or iodoacetic acid toxicity [25,26,27,28,32]. NaIO_3_-induced localized selective outer retinal damage in the porcine retina mimics that feature of human GA and represents a reproducible model that can be maintained over time [25], and also has utility to test the behavior of injected stem cell-derived RPE cells in the boundaries of the atrophic retina. FAF imaging was found to be a useful tool to detect the hiPSC-RPE cells in retinas. Although lipofuscin accumulates with age [52], the animals used in this study were too young to have an accumulation of this pigment, and FAF imaging was able to capture the hyper-autofluorescence emitted by GFP from the injected hiPSC-RPE cells, surrounded by the dark fundus of the retina.

Several studies have reported attempts at pre-clinical RPE cell therapy in the pig model, with experimental designs varying with regards to the cell delivery method employed (single cells [35] or sheets [33,34,53,54]), the species origin (swine [31,35,54] or human [27,33,34,53]), and the RPE cell source (pluripotent stem cells [33,34,35,53], adult [54], or fetal [27,31] RPE cells). To our knowledge, the present study is the first one to employ single-cell injection of human iPSC-derived RPE cells in pigs. Upon subretinal transplantation, the capacity of grafted cells to functionally integrate into the host retina relies on their survival and migratory capacity [55]. In line with this, our results show that transplanted RPE cells achieved high survival rates and organized forming an RPE monolayer in the presence of a healthy environment, such as in regions of the healthy retina and atrophy border. Compared to RPE sheets on scaffolds, we observed that the administration of cell suspension into the subretinal space of the eye offers advantages: (i) it is technically easier and less traumatic than the transplantation of RPE sheets or scaffolds, (ii) it reduces the chance to trigger an immune response or inflammation due to the presence of scaffolds’ products, (iii) it allows the injection of an accurate cell number in a localized area, and (iv) it enables better isolation, characterization, and cryopreservation, as well as quality control of the transplantable cell type. However, in atrophic areas where the environment is hostile and the rate of transplanted cell survival decreased, the transplantation of RPE sheets may increase their survival.

As expected, hiPSC-RPE cells failed to engraft properly as an epithelial-like structure within atrophic areas, and only three animals showed some isolated surviving cells. Although our animals were under immunosuppression, we cannot exclude the possibility that the combination of severe disruption of the RPE/BM layers together with the presence of xenogenic cells caused an immune response and graft rejection observed in some eyes, which is similar to the rejection observed by Sohn et al. [35]. Another possibility is the implication of choriocapillaris and the vascular endothelial growth factor (VEGF) in this model of GA, similar to the neovascular form of human AMD. Future studies on the VEGF levels in the healthy retina and in the GA before and after iPSC in our mode would be interesting to elucidate its role, if any, in the advanced stage of the dry form of AMD.

The RPE cells generated from hiPSC in this study have previously been shown to have specific RPE functions in vitro, such as phagocytosis of photoreceptor outer segments, ion transport, polarized factor secretion, membrane potential, and gene expression, similar to their native counterparts [56,57]. Extending our previous work on differentiation of hESC and hiPSC to RPE cells [58,59,60], here, we have further characterized differentiated RPE cells from human cord blood-derived iPSC, and shown that they exhibit cell properties, in vitro functions, and gene expression similar to native RPE cells. Our RPE confluent cultures expressed apical tight junctions and reached a TEER of >200 Ω·cm^2^, which is similar to that seen in vivo [56], suggesting a strong polarization and good tight junction integrity, important for their barrier function.

In the eye, RPE cells interact continuously with photoreceptor light-sensitive outer segments and are responsible for the phagocytosis of shed distal outer segments—a daily process that is essential for the renewal and survival of photoreceptors [61]. We showed here that hiPSC-RPE cells have the capacity to phagocytize POS in vitro. This is important to consider for the optimization of protocol conditions and the establishment of specific characteristics to ensure the transplantation of functional cells. We found that hiPSC-RPE cells indeed engrafted to some extent in the host healthy retina near the border of the atrophy in five animals and were able to form a mature quiescent long-term extensive epithelial-like layer in the healthy porcine retina in some animals. More importantly, several engrafted hiPSC-RPE cells showed numerous immunolabeled phagosomes, suggesting a potential interaction with host photoreceptors. This would support the rationale of using hiPSC-RPE injection as a regenerative therapy with the intention of assisting native RPE in decreasing the rate of atrophy progression.

Prior studies of transplanted hESC-RPE cells in a rabbit model of GA showed reduced pigmentation that was recovered over time [62,63]. In line with this, we confirmed that although the hiPSC-RPE cells used here acquired pigmentation in vitro, in the short-term integration period in porcine retinas, the cells lost their morphology and pigmentation according to their proliferative state, resembling the in vitro re-maturation process after cell expansion. However, at three months post-transplantation, RPE cell pigmentation and functional layer formation were re-established.

From a safety perspective, iPSC-based therapies must overcome the clinical hurdle of tumorigenicity, associated with the transplantation of undifferentiated cells or progenitors with increased genomic instability. In this regard, we first assessed the cell integrity and purity of our hiPSC-RPE cultures to exclude undifferentiated cells using a protocol of cell sorting and purification by extensive culture and passaging. Upon transplantation, there was no evidence of neoplastic or teratoma structures or any other adverse event in any of the transplanted porcine eyes studied, in accordance with other studies in different animal models and in humans [12,58,59,64]. Furthermore, there was no evidence of endophthalmitis, retinal edema, uveitis, epiretinal membrane, proliferative vitreoretinopathy, encapsulation, or retinal rupture in the immunosuppressed animals. One animal developed a retinal detachment due to persistence and enlargement of the bleb. Therefore, the current implantation subretinal transplant surgical technique appears to be safe and reproducible. Despite not performing vitrectomy and posterior hyaloid removal, epiretinal membrane formation was not an issue, which is usually the main complication of this type of subretinal transplantation.

Several human phase I/II clinical trials using RPE derived from hESC are in progress in patients under immunosuppression (e.g., NCT01344993). However, one important advantage of using hiPSC-derived cells would be the possibility to create HLA-haplotype banking of hiPSC (i.e., from umbilical cord banks) to cover most of the HLA types of the population [65], which would circumvent the need for immunosuppression in the elderly patients and would favor long-term transplanted cell survival. This might be a key advantage of using iPSC over hESC for the clinical translation of this therapy to a large population of patients. However, several strengths and limitations of the present study warrant further discussion. The major limitations of this study were the lack of a true animal model of AMD and the impossibility of quantitatively measuring the magnitude of the lesion induced with the NaIO_3_. Despite these obstacles, this model offers a quite selective outer retinal damage within a lesion with well-demarcated distinct boundaries, representing an optimal model to evaluate the feasibility of stem cell engrafting at the margins of the lesion. Our present study was also limited by the low number of animals used and the relatively short follow-up time. Testing more animals and over longer time periods will be necessary to evaluate the efficacy of the procedure, as will be including a control group that does not receive a RPE cell injection. These limitations notwithstanding, we believe that the results of our observational study clearly support the feasibility of hiPSC-derived RPE cell transplantation in pigs and set the foundation for future preclinical trials addressing efficacy.

In summary, we showed that subretinal NaIO_3_ is a robust model mimicking some of the features of human GA, and seems to be appropriate to test stem cell-based transplantation. Subretinal injection of hiPSC-RPE cell suspensions is a feasible approach with a high rate of long-term cell survival in areas of a healthy retina, at the borders of the atrophy. Additionally, we provided findings suggestive of iPSC-derived RPE integration, including phagosomes from retinal phagocytosis, which might underscore the use of these RPE cells to treat atrophic AMD in a supportive housekeeping role by improving the survival of overwhelmed RPE cells and photoreceptors at the boundaries of the atrophy, decreasing the rate of progression of the GA, and consequently delaying the onset of loss of visual function. Human phase I/II studies are needed as a next step to assess the safety and efficacy of these cells on visual function deterioration or lesion size progression in patients with GA secondary to AMD.

## 4. Materials and Methods

### 4.1. Cell Culture

Ethics review board and competent authority approval were obtained for this study (472 380 1; 12 July 2019). An hiPSC line derived from cord blood CD133+ cells, CBiPS30-4F-5 [66], was obtained from the Spanish Stem Cell Bank. hiPSC were maintained and expanded in mTeSR1 medium (Stem Cell Technologies, Vancouver, BC, Canada) on Matrigel^TM^ (Corning, NY, USA)-coated plates at 37 °C with 5% (*v*/*v*) CO_2_.

### 4.2. Generation of hiPSC Expressing GFP and Their Differentiation to RPE Cells

To facilitate the detection of RPE cells in the host retina, we first generated an hiPSC line constitutively expressing green fluorescent protein (GFP) by lentiviral transduction (Supplementary Materials and Methods). The hiPSC-GFP line was differentiated to RPE cells as previously described [58,59,60]. The hiPSC-GFP line was expanded on Matrigel-coated 10 cm plates in mTeSR1 medium until the colonies reached 60% confluence. Then, the medium was replaced with RPE medium (DMEM/F12 (Gibco, supplemented with 5% Knockout Serum Replacement (Gibco; Thermo Fisher Scientific, Inc., Waltham, MA, USA), 1% N2 (Gibco; Thermo Fisher Scientific, Inc., Waltham, MA, USA), 2% B27 (Gibco; Thermo Fisher Scientific, Inc., Waltham, MA, USA), 0.1 μM dexamethasone (Sigma Aldrich, St. Louis, MO, USA), 10 mM β-glycerolphosphate (Sigma Aldrich, St. Louis, MO, USA), 20 ng/mL human IGF-1 (R&D Systems, Minneapolis, MN, USA), and 10 mM nicotinamide (Sigma Aldrich, St. Louis, MO, USA) and changed every 2–3 days for 6–8 weeks. Pigmented areas were manually picked, dissected with 0.25% trypsin, and re-plated on Matrigel-coated plates. After 3–4 weeks of differentiation, when cells reached confluence, they were purified by incubation with 0.05% trypsin for 2 min to remove fibroblast-like cells. The remaining cells were disaggregated in TrypLE Select (Gibco; Thermo Fisher Scientific, Inc., Waltham, MA, USA), re-seeded on Matrigel, and maintained on RPE medium. Single hiPSC-derived RPE (hiPSC-RPE) cells were sorted on a Moflo XDP cell sorter (Beckman Coulter, Brea, CA, USA), and only GFP+ cells were expanded for injection (Supplementary Figure S1C,D). hiPSC-RPE cells were frozen and stored for later use at passages 2–4.

### 4.3. Immunocytochemistry

hiPSC-RPE cells were fixed in 4% paraformaldehyde for 20 min at room temperature, permeabilized, and blocked with tris-buffered saline (TBS) containing 0.5% (*v*/*v*) Triton X-100 and 6% (*v*/*v*) normal donkey serum (Sigma-Aldrich; St. Louis, MO, USA) for 1 h at room temperature. Primary antibodies were incubated at 4 °C overnight in phosphate-buffered saline (PBS), 0.1% (*v*/*v*) Triton X-100, and 6% (*v*/*v*) normal donkey serum, and secondary antibodies (1:500; Jackson Immunoresearch Laboratories, West Grove, PA, USA) were incubated in the same buffer at 37 °C for 2 h. Nuclei were stained with 4′,6-diamidino-2-phenylindole (DAPI) (Sigma-Aldrich; St. Louis, MO, USA) and cells were mounted in an anti-fading mounting medium. Confocal images were obtained on a TCS SP5 confocal microscopy (Leica Microsystems, Wetzlar, Germany) using a 40× or 63× oil immersion objective, and z-stacks were acquired at 1024 × 1024 pixels. Antibodies are listed in Supplementary Table S1.

### 4.4. Messenger RNA Expression by Quantitative Reverse-Transcriptase Polymerase Chain Reaction

Total RNA from hiPSC-RPE cells was isolated using TRIzol reagent (Sigma Aldrich, St. Louis, MO, USA), and cDNA was synthesized using the Cloned AMV First-Strand Synthesis Kit (both from Invitrogen, Carlsbad, CA, USA) [67]. Gene expression levels were analyzed by qRT-PCR (qPCR) reaction using SYBR green chemistry (Life Technologies, Carlsbad, CA, USA) and run on the ABI PRISM 7900HT platform (Applied Biosystems, Foster City, CA, USA) by a standard two-step amplification program: hot start activation (95 °C for 5 min), denaturing step (95 °C for 15 s), and annealing/extension step (60 °C for 30 s), repeated for 40 cycles. Primer sequences are listed in Supplementary Table S2. Threshold cycle (Ct) values were normalized to those of the housekeeping gene *GAPDH* and relative to hiPSC genes, and were expressed as 2^-ΔΔCt^ (log scale).

### 4.5. Transepithelial Electrical Resistance Measurements

hiPSC-RPE cells were cultured on Matrigel-coated Transwell filter inserts (Millipore, Burlington, MA, USA) in RPE medium until the complete formation of the epithelial monolayer. Electrical measurements were performed every 3 days for 3 weeks in biological triplicates using an epithelial voltohmmeter (EVOM; World Precision Instruments Inc., Sarasota, FL, USA). Transepithelial electrical resistance (TEER) values were corrected by subtracting values of a blank and multiplied by the cell growth area. Values were presented in Ω·cm^2^.

### 4.6. Electron Microscopy

For transmission electron microscopy (TEM), hiPSC-RPE cells were cultured on Matrigel-coated Transwell inserts and fixed with 2.5% (*w*/*v*) glutaraldehyde in 0.1 M cacodylate buffer, pH 7.2–7.4, for 2 h at 4 °C. Post-fixation was carried out in 1% (*w*/*v*) osmium tetroxide for 2 h at 4 °C, and samples were then dehydrated with an ascending ethanol series and embedded in epoxy resin. Ultrathin sections were examined in a Jeol 1011 transmission electron microscope (Jeol, Tokyo, Japan).

For scanning electron microscopy (SEM), RPE cells were cultured and fixed as for TEM. Fixed cells were dehydrated with an ascending ethanol series, dried in a critical point dryer, and metalized, and then examined in a Jeol JSM-6390LV scanning electron microscope (Jeol, Tokyo, Japan).

### 4.7. Photoreceptor Outer Segment Phagocytosis Assays

hiPSC-RPE phagocytosis activity was assessed by analyzing the binding and internalization of photoreceptor outer segments (POS) labeled with tetramethylrhodamine isothiocyanate (TRITC; Sigma-Aldrich Corp.St Louis, MO, USA), as described previously [68]. Unlabeled bovine POS were obtained from Fondation Voir et Entendre (Paris, France). POS were incubated with TRITC in labeling buffer containing 10% sucrose, 20 mM disodium phosphate (Sigma Aldrich, St. Louis, MO, USA), pH 7.2, and 5 mM taurine (Sigma Aldrich, St. Louis, MO, USA), and rotated for 1 h in the dark. Subsequently, POS-TRITC were washed twice with labeling buffer and once with DMEM/F12 medium, and were then resuspended in DMEM/F12. hiPSC-RPE cells were incubated with POS-TRITC for 4 h at 37 °C with 5% (*v*/*v*) CO_2_ in RPE medium, washed four times in PBS with Ca2+ and Mg2+, and then fixed in 4% (*w*/*v*) paraformaldehyde for 20 min at room temperature. Confocal images were taken with a TCS SP5 confocal microscope (Leica Microsystems, Wetzlar, Germany).

### 4.8. Animals, Preoperative Preparation, and Study Design

Experiments in pigs were approved by the Institutional Ethics Committee for Animal Experimentation and were carried out in accordance with the ARVO Statement for the Use of Animals in Ophthalmic and Vision Research. Ten healthy female miniature (mini) pigs (Sus scrofa domesticus) (provided by Specific Pig S.L., Barcelona, Spain) aged 7–8 months and 20–30 kg body weight were used in the study (preoperative preparation and medication are described in Supplementary Materials and Methods). The present study is a dose-escalating exploratory pre-clinical study to assess the feasibility of RPE cell injection. The nature of this study is observational and, to maximize the variables explored, we used 2 doses of NaIO_3_ and 2 concentrations of cells. Histology and immunostaining were performed and analyzed by researchers blinded to the sample experimental condition.

### 4.9. NaIO_3_ Subretinal Injection

To generate a pig model with features resembling some of those more characteristic of GA (damage of outer retinal layers with preservation of the inner layers, and lesions with well-defined distinct margins between the atrophic area and the healthy area), the procedure was based on a previous report that had defined the optimal NaIO_3_ concentration [25]. Degeneration of outer retinal layers was induced in the right eye of 10 minipigs through localized subretinal injection of NaIO_3_. To observe the behavior of the cells under different conditions of atrophy, we used three different NaIO_3_ concentrations: 0.01 mg/mL in pigs 1–7, and 0.1 mg/mL in pigs 8–10 (Table 1). Animals were placed in lateral recumbence with the snout slightly turned. The right eyes and the periocular skin were cleaned with povidone iodine solution diluted 1:10 in saline serum. Two blepharostats were placed to maximize the operating field and to avoid canthotomy, if possible. Two 23G valve trocars were inserted 1–1.5 mm posterior to the limbus, one for the 23G-endoilluminator and the other for the 25/41G-subretinal injection cannula connected to the ACCURUS^®^ Surgical System through an extension tube and a viscous fluid control system (all apparatus from Alcon Cusí S.A., Barcelona, Spain). No infusion canula was used. No vitrectomy was performed. Atrophy was induced by subretinal injection of 0.1–0.2 mL NaIO_3_ between the arcades using a subretinal injection cannula (25/41G DeJuan; Bausch + Lomb S.A., Madrid, Spain) near and superior to the optic nerve head. Trocars were removed without sutures of the self-sealing sclerotomies, and the eye pressure was checked.

### 4.10. Subretinal Injection of hiPSC-RPE Single Cell Suspension

hiPSC-RPE subretinal transplantation was performed 30 days after the lesion induction with NaIO_3_, when outer retinal atrophy was well-established. The cultured hiPSC-RPE cells were disaggregated with TrypLE Express (Invitrogen, Carlsbad, CA, USA), washed 3 times in PBS, and resuspended in DMEM/F12 medium. A subretinal bleb of 0.1–0.2 mL with a suspension of hiPSC-RPE cells containing 2.5 × 10^5^ or 3.3 × 10^5^ cells (Table 1) was created with an injection cannula (25/41G DeJuan; Bausch + Lomb). Retinotomy and bleb induction were performed in the healthy margins and within areas of the degenerative retina, such that it was also covered with cells. Cell survival after passing through the cannula was tested in vitro by flow cytometry using Propidium Iodide Cell survival rates that were similar to those of noninjected cells (Supplementary Figure S1E,F). In addition, 7 days before the cell transplantation and until euthanasia, animals were immunosuppressed by a daily oral administration of cyclosporine (Sandimmun Neoral, 15 mg/kg; Novartis Farmacéutica S.A., Basel, Switzerland) and prednisone (Prednisone Kern Pharma, 1 mg/kg; Kern Pharma S.L., Barcelona, Spain).

### 4.11. Postoperative Follow-Up

All animals were periodically assessed with a complete ophthalmic examination, summarized in Table 1. Prior to hiPSC-RPE cell subretinal injection, all animals were evaluated to assess the atrophy and rule out complications. After subretinal injections, animals 1 and 3–9 were evaluated at days 30, 60, and 90. Animal 2 died two weeks after cell injection for reasons unrelated to the study, but the eyes were successfully conserved. Animal 10 developed a retinal detachment due to a persistent enlargement of the bleb and was euthanized. Animal 1 was also analyzed in vivo at four, five, and six months to assess long-term cell survival. Animals 5–7 were further evaluated at day fifteen after hiPSC-RPE subretinal injection to discard inflammation or immunological response in the first two weeks. In all cases, the ophthalmological examinations were performed under sedation and the untreated contralateral eyes were considered as controls. Assessment of the retinas was carried out with infrared (IR), fundus autofluorescence (FAF; excitation filter at 488 nm; barrier >500 nm), and spectral-domain optical coherence tomography (SD-OCT) imaging using the Spectralis HRA+OCT^®^ device (Heidelberg Engineering, Heidelberg, Germany). All SD-OCT exams were performed with tracking activated from baseline if possible. Euthanasia was carried out under sedation and by applying an intravenous sodium pentobarbital overdose at 80 mg/kg.

### 4.12. Histology and Immunohistochemistry

Porcine eyes were enucleated, fixed in 4% (*w*/*v*) paraformaldehyde, and processed as described [25]. Tissue-Tek optimal cutting temperature compound (Sakura Finetek, Torrance, CA, USA -embedded retinal cross-sections (20 µm) were analyzed by hematoxylin and eosin (H&E) staining following a standard protocol. Images were taken with a Leica DM6000 microscope (Leica Microsystems, Wetzlar, Germany).

Prior to the immunohistochemistry, porcine retinal transverse cryosections were incubated with 0.1 M citrate buffer (pH 6.0) for 1 h at 60 °C to expose antigens. Immunohistochemistry of cryosections was performed as described above in the immunocytochemistry section.

### 4.13. Image Processing

Longitudinal analysis of fundus images was performed by aligning IR and FAF images to create overlapped images using FIJI software (ImageJ software; NIH, Bethesda, MD, USA). Images were processed using LAS AF software (Leica Microsystems, Wetzlar, Germany), FIJI software, and/or Photoshop (Adobe Systems Inc., San Jose, CA, USA).

## Figures and Tables

**Table 1:** List and details of procedures and findings of each animal in this study.

Animal	NaIO_3_ dose	In vivo	hiPSC-RPE Cells Injected	Follow-up	Evidence of hiPSC-RPE in vivo		Post-Mortem Analysis	
	(mg/mL)			Months	FAF	SD-OCT	Affected Layers in the Atrophy	GFP+ Cells (Area and Layers)
1	0.01	Selective atrophy of ONL	3.3 × 10^5^	6	Yes	Yes	ONL, RPE	Healthy retina, atrophy (RPE)
2	0.01	Selective atrophy of ONL	3.3 × 10^5^	0.5 *	ND	ND	ONL, RPE	Healthy retina, atrophy and border (RPE)
3	0.01	Selective atrophy of ONL	3.3 × 10^5^	3	No	No	ONL, RPE	No cells
4	0.01	Selective atrophy of ONL	3.3 × 10^5^	3	No	No	ONL, RPE	No cells
5	0.01	Selective atrophy of ONL	2.5 × 10^5^	3	Yes	Yes	ONL, RPE	Healthy retina and vitreous
6	0.01	Selective atrophy of ONL	2.5 × 10^5^	3	Yes	Yes	ONL, RPE	Healthy retina (RPE)
7	0.01	Selective atrophy of ONL	2.5 × 10^5^	3	Yes	Yes	ONL, RPE	Atrophy (INL, RPE), border and vitreous
8	0.1	Marked atrophy and thinning of all retinal layers	3.3 × 10^5^	3	No	No	INL, ONL, RPE	No cells
9	0.1	Marked atrophy and thinning of all retinal layers	3.3 × 10^5^	3	Yes	Yes	INL, ONL, RPE	Healthy retina and border (RPE)
10	0.1	Retinal detachment	ND	ND	ND	ND	ND	ND

*: Exitus for reasons unrelated to the study before the first follow-up after the cell injection. ND: Not done; FAF: fundus autofluorescence; ONL: outer nuclear layer; INL; inner nuclear layer; SD-OCT: spectral-domain optic coherence tomography; RPE: retinal pigmented epithelium; GFP; green fluorescent protein.

## Data Availability

The data presented in this study are available in Supplementary Materials.

## Supplementary Materials

The following are available online at https://www.mdpi.com/article/10.3390/ijms221910497/s1.
