# Supplementary material for: Transplantation of Human Induced Pluripotent Stem Cell-Derived Retinal Pigment Epithelium in a Swine Model of Geographic Atrophy
Source: Int J Mol Sci. 2021 Sep 28;22(19):10497. doi: 10.3390/ijms221910497 (PMC8508834; doi:10.3390/ijms221910497)
Supplement: Supplementary file 1 [file ijms-22-10497-s001.zip › ijms-1405296-supplementary.pdf]

SUPPLEMENTARY MATERIALS

MATERIALS AND METHODS

SparQ-GFP lentiviral production and hiPSC transduction

HEK293T (human embryonic kidney 293T) cells were cultured in Dulbecco's modified Eagle's medium (DMEM) supplemented with 10% FBS (Hyclone), 1% Glutamax, 1% non-essential amino acids, 100 units/ml penicillin and 100 µg/ml streptomycin, at 37 °C under humidified air containing 5% CO_2. Lentiviral particles were produced using a 2nd generation lentiviral system. Briefly, HEK293T cells were transfected with SparQ-GFP (System Biosciences), psPAX2 and pMD2.G (Addgene) at a ratio of 1:0.75:0.25 µg DNA ratio respectively, using Fugene 6 (Roche). After 16 h, medium was replaced with fresh medium. Virus-containing medium was collected after 24 h and 48 h and viral particles were concentrated by ultracentrifugation. The CBiPS30-4F-5 line was transduced with 1000-fold diluted SparQ-GFP viral particles in the presence of 10 µg/ml of polybrene in mTeSR1 medium. After 24 h, cells expressed GFP under the EF1 promoter and the medium was changed every other day. Cells were disaggregated and GFP+ clones were selected.

Flow cytometry analysis

hiPSC, hRPE and hRPE-GFP cell cultures were dissociated into single cells using TrypLE Select, neutralized and resuspended in cold PBS and filtered through a 30-µm filter to remove clusters and debris. hRPE-GFP single cells were sorted for GFP using the MoFlo® XDP cell sorter (Beckman Coulter). Viable cells were gated by their forward and side scatter characteristics, and gates were set to sort positive and negative cell populations. To test cell viability after passing through the cannula, hRPE-GFP+ cells were stained with propidium iodide (mg/mL) and analyzed by flow cytometry using a Gallios flow cytometer (Beckman Coulter) to measure cell apoptosis, which was compared with that of control cells. To assess hRPE-GFP cell culture purity, hiPSC and hRPE-GFP single cells were cells were stained with the Live/Dead fixable Violet (Invitrogen) for 30 min following by 2 % PFA fixation. Cells were incubated with Alexa Fluor 647 Mouse Anti-Human TRA-1-60, PE Mouse Anti-Human CD140b, and PE Mouse Anti-Human CD59 diluted in 2% normal donkey serum (Thermo Fisher Scientific) for 30 min protected from the light at room temperature (antibodies are listed in Supplemental Table S1). Stained cells were analyzed using a LSR Fortessa Cell analyzer (BD Biosciences) equipped with 405, 488, 561, and 640 nm lasers. Flow cytometry analysis was performed by gating out death cells, doublets and the debris. Unstained cells and secondary antibodies were used as negative controls and hRPE cells were used as a negative control for GFP. Analysis of the data was carried out using the FCS Express software (BD Biosciences).

Karyotyping

The hiPSC-GFP line was karyotyped to evaluate its genomic integrity. Karyotyping (Ambar, Barcelona) was performed on G-banded metaphase chromosomes following standard procedures. A minimum of 20 metaphases were examined.

Terminal dUTP Nick End Labeling (TUNEL) Staining

Porcine eye cryosections (20–40 um) were assayed by TUNEL staining of fragmented DNA using the In Situ Cell Death Detection Kit, TMR red (Roche) with some modifications. To amplify the fluorescent signal, retinal cryosections were incubated with a goat anti-rhodamine antibody overnight at 4ºC followed by a Cy3-conjugated anti-goat IgG for 2 h at room temperature. Nuclei were stained with DAPI. Negative control reactions omitted the enzyme step in the reaction and positive controls were established by treating tissue sections with DNase I.

Preoperative preparation and medication

Animals were premedicated with an intramuscular injection of atropine (0.04 mg/kg; B. Braun Medical S.A., Barcelona, Spain) in combination with dexmedetomidine (Dexmopet, 0.03 mg/kg; Fatro Ibérica S.L., Barcelona, Spain), midazolam (0.3mg/kg; Laboratorios Normon S.A., Madrid, Spain) and butorfanol (Alvegesic, 0.3 mg/kg; Dechra S.L., Barcelona, Spain). Prior to surgery, animals were anesthetized through intravenous injection of propofol (Propovet multidosis, 0.5–1mg/kg; Zoetis S.L., Madrid, Spain), and were endotracheally intubated to maintain general anesthesia with isoflurane (Isoflo; Zoetis S.L.) with a minimal alveolar concentration of 2–4% and 100% oxygen. Additionally, the pigs received a complementary medication of anti-inflammatory (meloxicam 0.4 mg/kg; Metacam; Boehringer Ingelheim Vetmedica GmbH S.A., Ingelheim/Rhein, Germany) and antibiotic enrofloxacin (Baytril, 2.5 mg/kg; Bayer Hispania S.L., Barcelona, Spain) treatments through intramuscular injections. Both pupils were dilated with 2–3 drops of phenylephrine (Colicursí phenylephrine, Novartis Farmacéutica S.A., Barcelona, Spain) and tropicamide (Colicursí tropicamida, Novartis Farmacéutica S.A.) at 100 mg/ml. After surgery, the isoflurane dose was reduced and animals were extubated when breathing reflex was detected. Topical administration of an anti-inflammatory and antibiotic ointment (chloramphenicol, 0.5 mg/g and dexamethasone, 10 mg/g) was applied on the study eyes after surgery and twice a day during 10 days. Euthanasia was carried out under sedation and applying an intravenous sodium pentobarbital overdose at 80 mg/kg.

SUPPLEMENTARY TABLES

Table S1. Antibodies used for immunochemical analyses.

**Name**	**Comercial house and reference**	**Dilution**
Alexa Fluor 647 Mouse Anti-Human TRA-1-60	BD Biosciences, 560850	0.5 µl
Bestrophin-1	Santa Cruz Biotechnology, sc-32792	1:25
GFAP	Dako, Z0334	1:500
Ku80	Cell Signaling, 2180	1:200
MITF	Santa Cruz Biotechnology, sc-56725	1:25
NANOG	R&D Systems, AF1997	1:25
OCT4	Santa Cruz, sc-5279	1:25
OTX2	Santa Cruz Biotechnology, sc-30659	1:25
PAX6	Covance, PRB278P	1:100
PE-conjugated CD140b	BD Biosciences, 558821	2 µl
PE-conjugated CD59	BD Biosciences, 560953	2 µl
PKC-alpha (H-7)	Santa Cruz, sc-8393	1:50
Recoverin	Millipore, AB5585	1:500
Rhodopsin	Sigma, O4886	1:500
RPE65	Novus Biologicals, NB100-355	1:100
SOX2	ABR, PA1-16968	1:100
SSEA4	Hybridoma Bank, MC-813-70	1:2
TRA-1-60	Millipore, MAB4360	1:100
ZO1	Millipore, AB2272	1:100

Table S2. Primer sequences used for qRT-PCR analysis.

Gene	Forward	Reverse
CRALBP	CCTCTCCTCAACTGTCCTG	CCCTCCTTTATTACCCATCCC
GAPDH	GTCAGTGGTGGACCTGACCT	AGGGGAGATTCAGTGTGGTG
OCT3/4	GTTCTTCATTCACTAAGGAAGG	CAAGAGCATCATTGAACTTCAC
OTX2	GACCACTTCGGGTATGGACT	TGGACAAGGGATCTGACAGT
PEDF	AGATCTCAGCTGCAAGATTGCCCA	ATGAATGAACTCGGAGGTGAGGCT
SIL	GTTGATGGCTGTGGTCCTTG	CAGTGACTGCTGCTATGTGG
TYR	ACTTACTCAGCCCAGCATC	GGTTTCCAGGATTACGCC

FIGURES

FIGURE S1

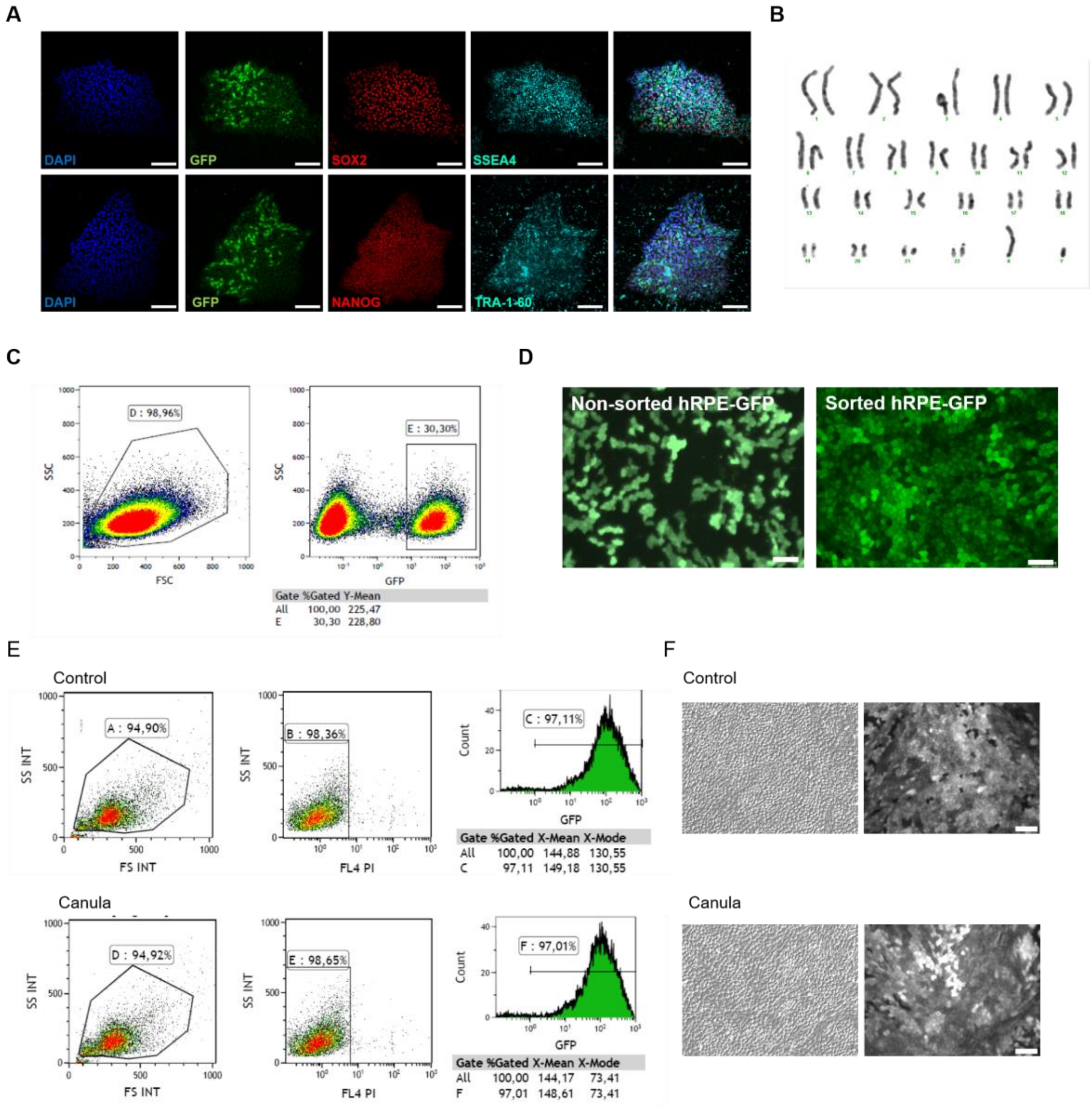

Figure S1: Characterization of CBiPS30-4F-5 human stem cell line-derived retinal pigment epithelial cells (RPE) expressing GFP. (**A,B**) Characterization of the transduced CBiPS30-4F-5-GFP clone. (A) hiPSC colonies expressed pluripotency markers SOX2, SSEA4, NANOG and TRA-1-60 (scale bars: 100 µm) and (**B**) maintained normal 46, XY karyotype. (C,D) Fluorescence-activated cell sorting of differentiated hiPSC-RPE-GFP cells in culture. (**C**) Forward *versus* side scatter plot of hiPSC-RPE cell population shows an homogeneous distribution, and side scatter *versus* GFP fluorescence intensity (in abscissas) shows the population considered positive (highlighted in a square). (**D**) hiPSC-RPE cells in culture before and after cell sorting. Scale bar: 75 µm. (**E,F**) Viability test of hiPSC-RPE cells after passing through the subretinal injection cannula (diameter 23/38G). (**E**) Flow cytometry quantitative analysis plots of side scatter intensity *versus* propidium iodide show excellent cell viability rate (98.65%) similar noninjected cells (98.36%). (F) After passing through the cannula, hiPSC-RPE cells were not damaged and remained viable after 10 days in culture. Scale bar: 75 µm. Similar results were obtained using a 25/41G subretinal injection cannula (not shown).

FIGURE S2

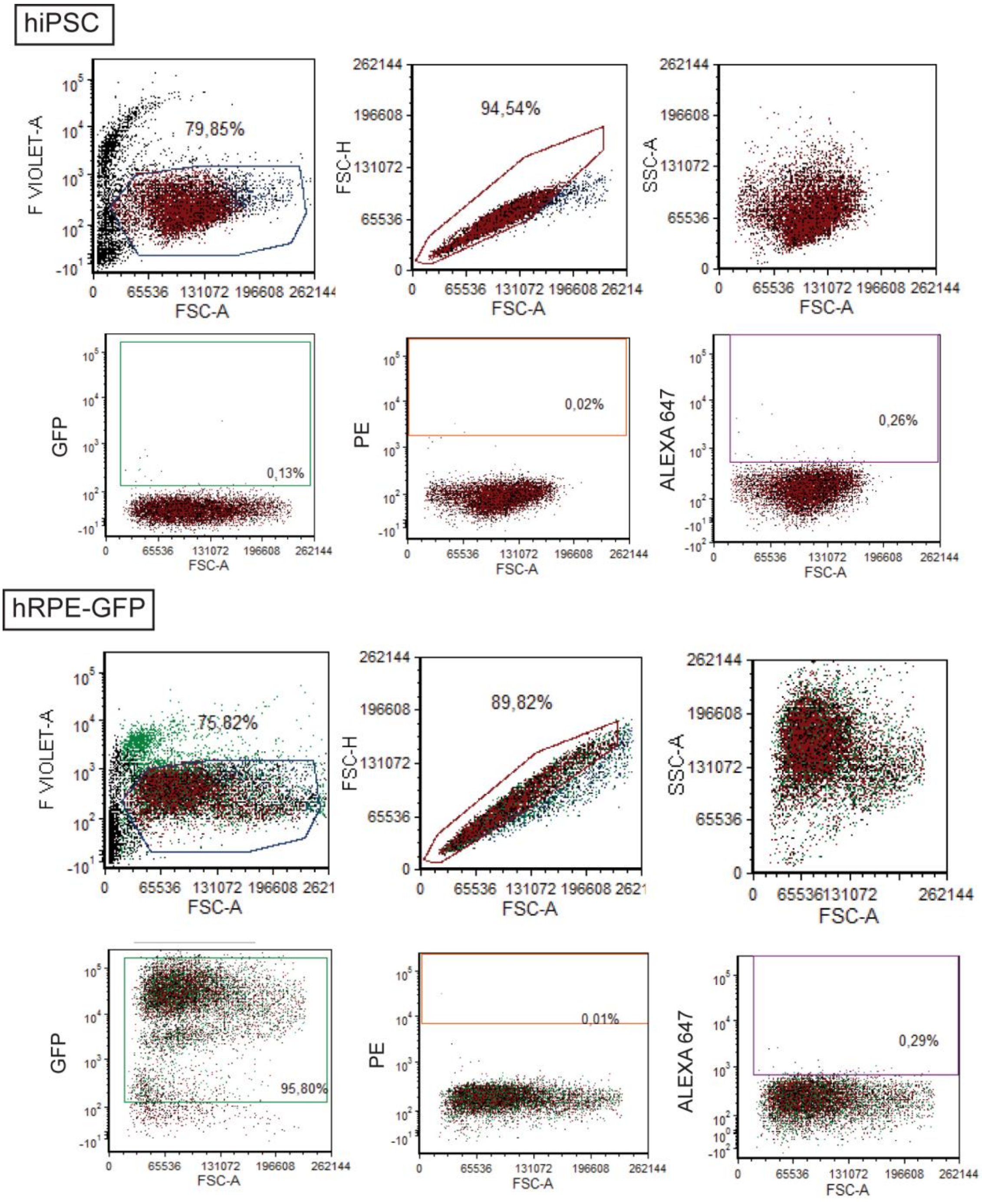

Figure S2: Flow cytometry strategy. Dot plot showing flow cytometry strategy indicating the live cells, dead cells, doublets and debris in hiPSC and hRPE-GFP cells. Unstained cells and specific isotype were used as negative controls.

FIGURE S3

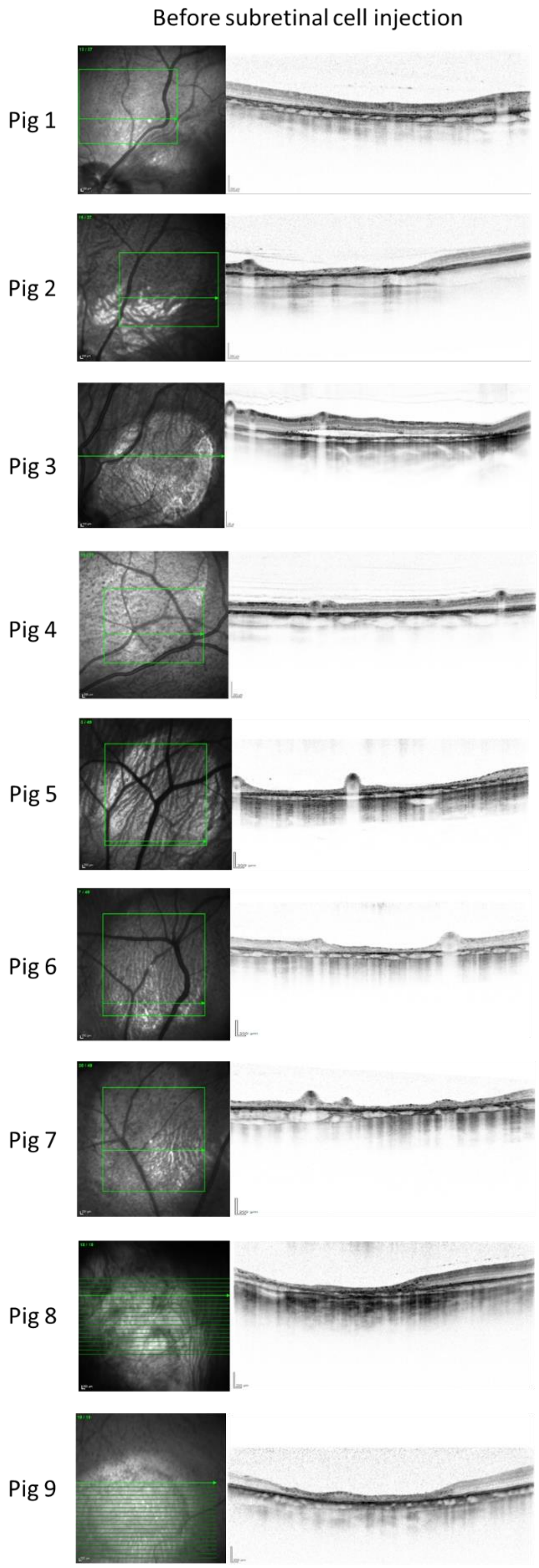

Figure S3: In vivo visualization of retinal atrophy lesions. SD-OCT guided by infrared fundus of all eyes at day 30 just before hiPSC-RPE subretinal cell injection, showing different degrees of selective outer retinal atrophy.

FIGURE S4

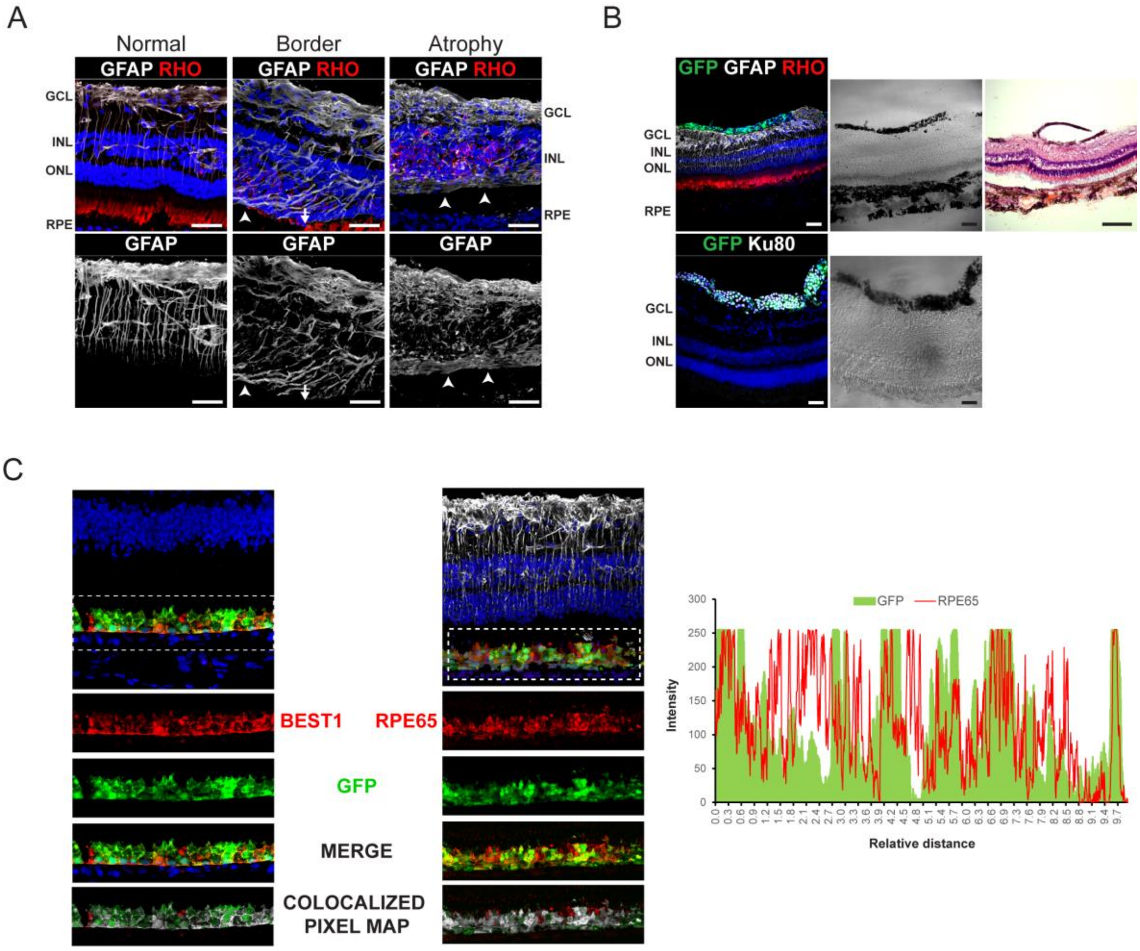

Figure S4: Characterization of retinal atrophy and transplanted hiPSC-RPE cells. (**A**) Immunostaining with GFAP (astrocytes) shows reactive changes in glial cells associated with retinal degeneration in the atrophic area. Photoreceptor cell loss is shown by rhodopsin (RHO) marker. Arrow indicates the atrophic border and arrowheads indicate glial membrane-like structures. Scale bars: 50 µm. (**B**) Immunofluorescent labeling of hiPSC-RPE cells located in the vitreous, stained with GFAP, Ku80 (human nuclear antigen) and RHO, accompanied with the corresponding bright field images. Right panel shows hematoxylin and eosin staining of pigmented hiPSC-RPE attached to the GCL in the vitreous site. Scale bars: 100 µm in top panels and 50 µm in bottom panels. (**C**) Colocalization analysis of GFP and RPE65 signals and GFP and BEST1 signals (from Fig. 6D) shown in the colocalized pixel maps. Plot profile shows GFP and RPE65 signal intensities across the layer (right). The fluorescent intensity was quantified using Image J software.
